# Titin is a nucleolar protein in neurons

**DOI:** 10.21203/rs.3.rs-4000799/v1

**Published:** 2024-03-04

**Authors:** BreAnna Cameron, Lauryn Torres-Hernandez, Virginia Lynne Montague, Karen A. Lewis, Heidi Smith, James Fox, Xueshui Guo, Robert G. Kalb, Lynn George

**Affiliations:** 1 Department of Biological and Physical Sciences, Montana State University Billings, Billings, MT.; 2 Department of Chemistry and Biochemistry, Texas State University, San Marcos, TX.; 3 Center for Biofilm Engineering and Department of Microbiology and Cell Biology, Montana State University, Bozeman, MT.; 4 Animal Resources Center, Montana State University, Bozeman, MT.; 5 Les Turner ALS Center – Feinberg School of Medicine, Northwestern University, Chicago, IL.

## Abstract

Titin is the largest protein produced by living cells and its function as a molecular spring in striated muscle is well characterized (1, 2). Here we demonstrate that titin isoforms in the same size range as found in muscle are prominent neuronal proteins in both the central and peripheral nervous systems, including motor neurons in the spinal cord and brain. Within these neurons, titin localizes to the dense fibrillar component of the nucleolus, the site of ribosomal RNA biogenesis and modification, and a critical site of dysfunction in neurodegenerative disease (3–5). Additionally, we show that the levels of both titin mRNA and protein are altered in the spinal cord of SOD1^G93A^ mice, a commonly used model of amyotrophic lateral sclerosis, indicating that titin mediated nucleolar events may in fact contribute to the pathobiology of disease.

With a contour length greater than a micron, titin is nearly twice the size of any other polypeptide known (1). Accordingly, the transcript encoding human titin, *TTN,* is also colossal, comprising over 300,000 nucleotides, and predicted to take more than an hour to transcribe (6). *TTN’s* 363 exons have a coding potential of up to 38,138 residues, or a protein 4200 kDa in size. Although there are potentially thousands of different titin isoforms, only a handful have been functionally characterized. The 3700 kDa N2A isoform and slightly smaller N2B cardiac isoforms are abundant in vertebrate striated and cardiac muscle, respectively, where they provide passive elasticity within the sarcomere (7).

After detecting titin in a neuronal proteomics study (8), we investigated the possibility that titin may in fact function as a *bona fide* neuronal protein. Here we demonstrate that titin is indeed present in numerous neuronal subtypes including alpha motor neurons, and that it localizes to the dense fibrillar component (DFC) of the nucleolus, the site of ribosomal RNA (rRNA) biogenesis and processing. Given the contributions of nucleolar stress to amyotrophic lateral sclerosis (ALS) (9–12), and that mutations in the gene encoding titin (*TTN*) are associated with a rapid functional decline in ALS (13), we posit that titin may be a previously unrecognized piece in the complex puzzle of neurodegenerative disease.

## Results

### Titin is present in the embryonic and adult nervous system

**Figure 1.**
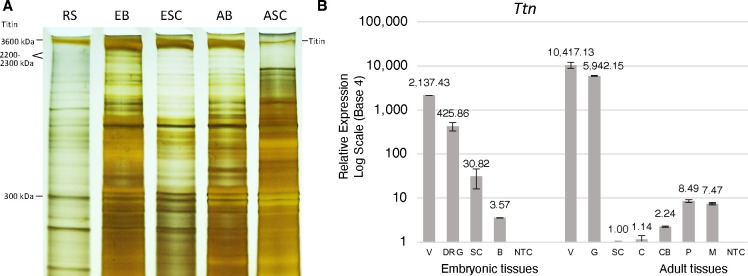
Titin expression in the embryonic and adult nervous system. (A) PAGE) and silver staining of spinal cord (SC) and brain lysates from embryonic and adult mice reveals bands in a the 3000 – 3700 kDa range. Titin isoform N2A from rabbit soleus (RS) (3600 kDa) is included as a size standard (18). Bands in the 2200–2300 kDa range in the RS lane are titin degradation products (19). (B) RT-qPCR shows *Ttn* expression levels in embryonic and adult nervous system tissues relative to muscle. EB, embryonic brain; ESC, embryonic spinal cord; AB, adult brain; ASC, adult spinal cord; V, ventricle; B, brain; NTC, no template control; G, gastrocnemius muscle; C, cerebral cortex; CB, cerebellum; P, pons; M, medulla.

We detected titin in a previous proteome analysis of embryonic dorsal root ganglia (DRG) (8). To further investigate the possibility that titin may function in the nervous system, we first queried the available literature and various gene and protein expression databases. Surprisingly, both *TTN* transcript and protein have been detected in multiple neuronal tissues including mouse brain and spinal cord, human brain, and human DRG (8, 14–17). Next, polyacrylamide gel electrophoresis (PAGE) was performed using both embryonic and adult mouse nervous system tissues and the titin N2A band from rabbit soleus muscle as a size standard (18). Bands in the 3000–3700 kDa range were present in all samples (Fig. 1A), a size unique to titin isoforms.

RT-qPCR was next performed to further investigate murine *Ttn* expression in neurons, and to provide a more quantitative assessment of its expression compared to muscle. As shown in Figure 1B, *Ttn* transcript is approximately 5-fold more abundant in the embryonic ventricle compared to the DRG, and approximately 700-fold more abundant in adult gastrocnemius muscle compared to the adult pons. Adult spinal cord showed the lowest levels of *Ttn* message, mirroring its protein levels in the adult spinal cord (Fig. 1A,B).

### Titin is present in the neuron nucleus

**Figure 2.**
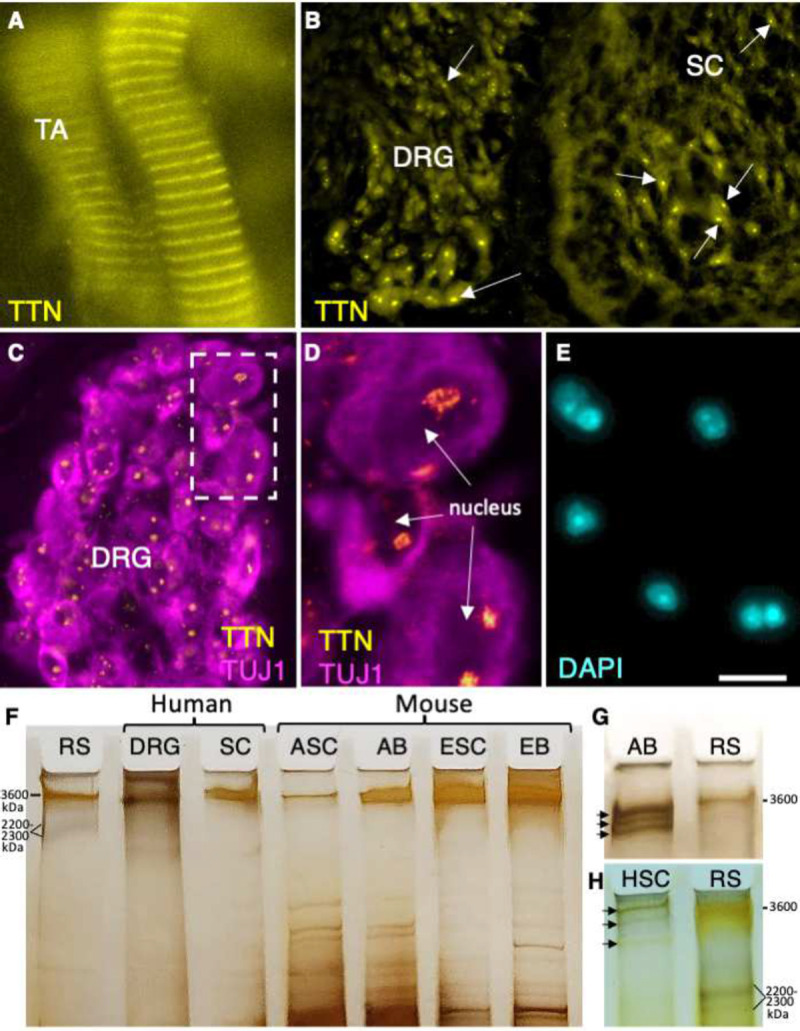
Titin localizes to the neuron nucleus. (A) Anti-titin antibody 27867-1-AP recognizes native titin in the adult mouse tibialis anterior (TA). (B-D) IHC using antibody 27867-1-AP on lumbar cross sections of an E17.5 mouse embryo shows punctate immunostaining within the nucleus of DRG and spinal cord (SC) neurons. (D) shows an enlargement of the boxed region in (C). (E) Nuclei isolated from an adult mouse spinal cord used for PAGE analysis. (F) PAGE analysis of nuclear extracts from mouse and human nervous system tissues generates bands in the 3000–3700 kDa size range. Titin isoform N2A (3600 kDa) from rabbit soleus (RS) (first lane) is shown as a size standard. (G,H) With longer run times, three bands are visible in lysates from adult brain and human SC. EB, embryonic brain; ESC, embryonic spinal cord; AB, adult brain; ASC, adult spinal cord. TUJ1, pan neuronal marker (Beta-III tubulin); DAPI, nuclear marker. Bar = 7.5 um in (A), 45 um in (B), 15 um in (C), 6 um in (D), 35 um in E.

Fluorescent immunohistochemistry (IHC) was next performed to investigate the nervous system cell types that might express titin and to analyze its localization within the cell. Anti-titin antibody 27867-1-AP (Proteintech) shows robust immunoreactivity in tissue sections of mouse muscle. Figure 2A highlights titin’s spiral architecture in the sarcomere, confirming 27867-1-AP recognizes native titin. Since embryonic DRG were used for our previous proteome investigations, tissue sections containing DRG from embryos of the same gestational age (E17.5) were examined first. Robust, punctate spots were visible in the nucleus of neurons within the DRG and the spinal cord ventral horn, with larger neurons exhibiting larger puncti (Fig. 2B–D).

**Figure E1.**
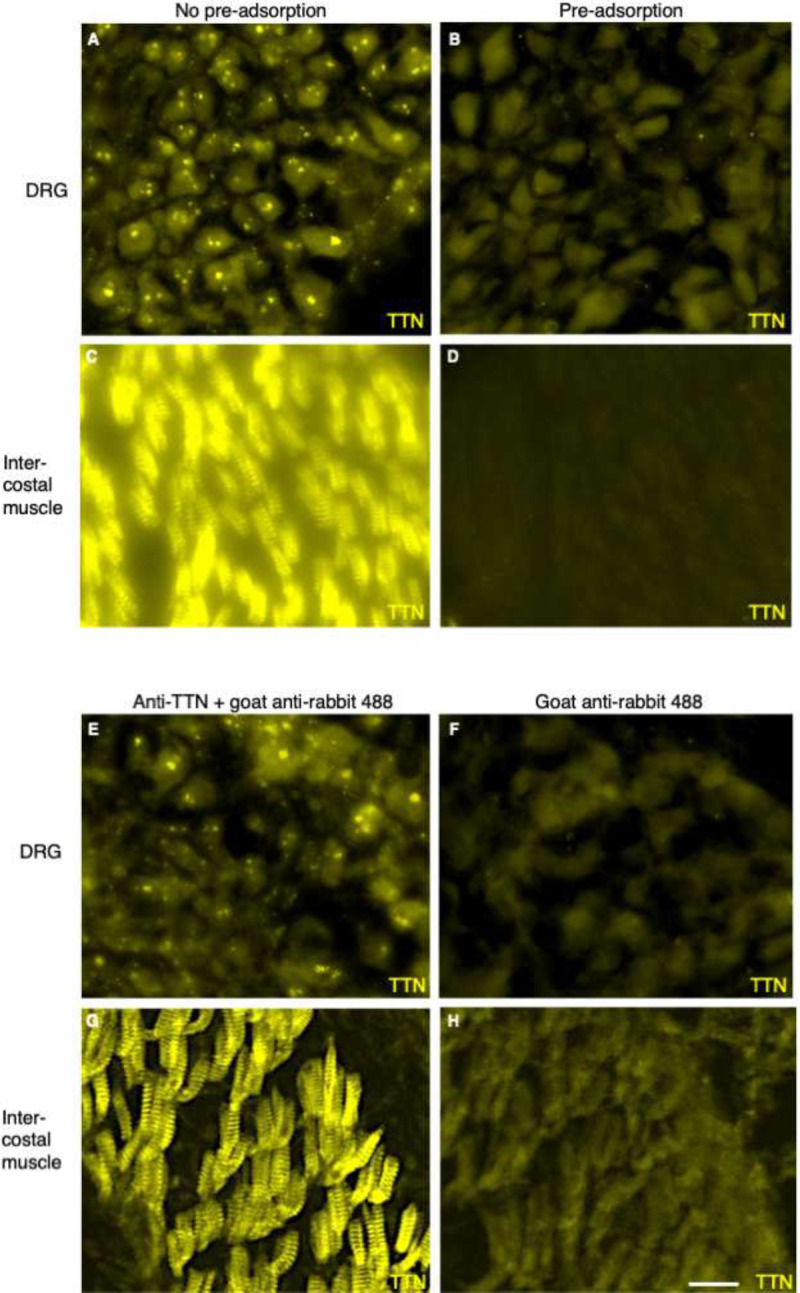
Negative Control experiments. (A-D) Preadsorption with titin fusion peptide Ag27496 completely blocks immunostaining of both DRG and intercostal muscle by anti-titin antibody 27867-1-AP (Proteintech). (E-H) No fluorescence is observed in either the DRG or intercostal muscle when IHC is performed in the absence of anti-titin antibody 27867-1-AP. DRG, dorsal root ganglion. Bar = 15 um in (A,B,E,F); 28 um in (C,D,G,H).

Anti-titin antibody 27867-1-AP was raised against the human titin Ig-like domain 149 and likely recognizes Ig-like domain 142 in mouse. This region of mouse titin shares 80% identity (186/232 residues) with the fusion peptide used to raise the antibody (Proteintech, Ag27496). To further test the specificity of the antibody, a pre-adsorption step was performed using titin peptide Ag27496, followed by IHC on tissue sections of muscle, spinal cord and DRG. This pre-adsorption step completely blocked the spiral labeling of muscle and the punctate staining in neuronal cells (Fig. E1A–D), again indicating that 27867-1-AP binds titin. Finally, no fluorescence was observed when IHC was performed in the absence of primary antibody (Fig. E1E–H).

To explore the possibility that in addition to titin, antibody 27867-1-AP may be recognizing a similar, but non-titin epitope, a blast search against all known *Mus musculus* proteins was performed using the Ag27496 peptide sequence. The top two hits landed on titin isoforms N2-B and N2-A, showing 80% amino acid identity and 99% coverage. The next 15 highest ranking hits were also titin isoforms with percent identities ranging from 68 to 80%. The highest scoring non-titin protein (37% identity, 49% coverage) was obscurin, a non-nuclear protein. These combined data strongly indicate that the nuclear protein recognized via IHC by antibody 27867-1-AP is titin.

As a second approach for investigating titin’s localization within the cell, we isolated nuclei from the same nervous system tissue sources as shown in Figure 1, as well as from human spinal cord and DRG (Fig. 2E) and used PAGE to again probe for titin’s presence. In agreement with our IHC results showing nuclear localization, bands in the titin size range were detected in these nuclear extracts (Fig. 2F). Titin’s size makes its transfer to a membrane particularly challenging. Thus, to confirm their identity, bands corresponding to adult mouse brain and rabbit soleus were excised from the gel and analyzed via LC-MS. As shown in Table E1, these extremely large bands contain titin. Additionally, increased run times resolved three bands in the 2000–3600 kDa range in both brain and spinal cord lysates including human spinal cord (Fig. 2G,H).

### Titin localizes to the nucleolar dense fibrillar component

**Figure E2.**
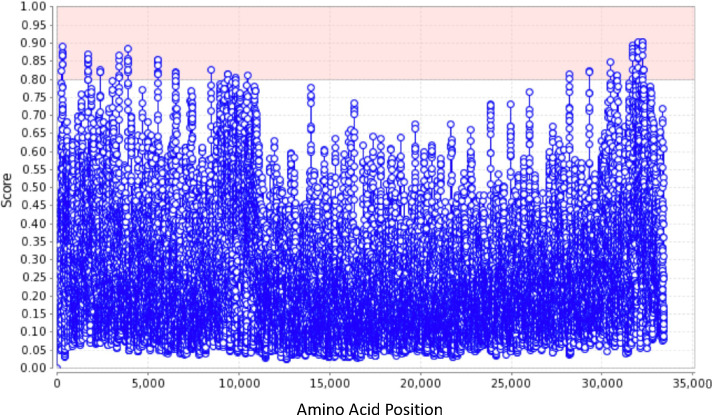
Nucleolar localization sequence predictions for human titin. Plotted by the online Nucleolar localization sequence Detector tool NoD (http://www.compbio.dundee.ac.uk/www-nod/). Scores above 0.80 (pink shaded region) qualify as positive hits.

**Figure 3.**
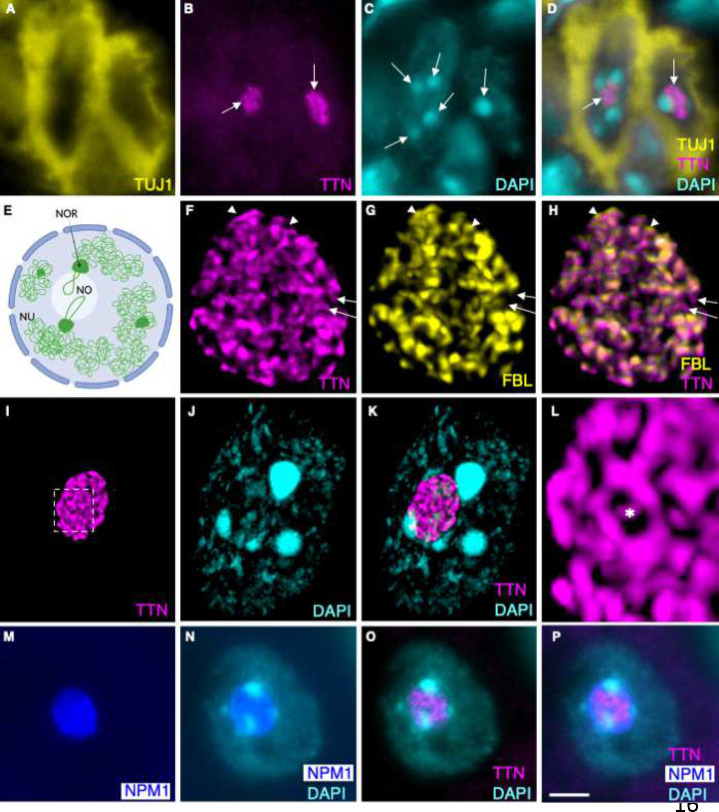
Titin localizes to the dense fibrillar component of the neuron nucleolus. (A-D) E17.5 embryonic DRG. Titin (arrows in B and D) localizes to the nucleolar region circumscribed by DAPI+ NORs (arrows in C) in TUJ1+ peripheral neurons (D). (E) Schematic illustrating NORs. Nucleolus (NO, white); nucleus (NU, light blue). Chromatin is green. Created with BioRender.com. (F-P) Alpha motor neurons in P21 mouse spinal cord. (F-H) LSCM shows that titin and FBL are tightly intertwined within the DFC. Arrows show regions of titin+, FBL-negative immunoreactivity. Arrowheads show regions of titin-negative, FBL+ immunoreactivity. (I-L) LSCM shows that titin exhibits a reticulate pattern, circumscribed by DAPI+ NORs. L shows a zoom of the boxed region in I, with asterisk centered in a single ring-shaped module of the DFC. (M-P) The NPM1+ granular component surrounds the TTN+ DFC. TUJ1, Beta-III Tubulin (neuronal marker); FBL, Fibrillarin; NPM1, Nucleophosmin. Bar = 4 um in (A-D), (M-P); 1 um in (F-H); 2 um in (I-K); 375 nm in (L).

The punctate pattern of titin immunostaining within a confined region of the neuron nucleus suggested that titin may localize to a specific subnuclear compartment. To further explore this possibility, IHC for titin was combined with 4′,6-diamidino-2-phenylindole (DAPI), a nuclear stain that binds AT rich regions of DNA. Here, titin appeared juxtaposed between bright DAPI+ spots within the fainter DAPI+ nucleus (Fig. 3A–D). Intense DAPI+ puncta mark chromocenters that form perinucleolar heterochromatin and contain nucleolar organizer regions (NORs) that localize around the perimeter of the developing nucleolus (Fig. 3E) (20). The juxtaposition of titin between NORs raised the possibility that titin may localize to the neuron nucleolus. IHC for titin and a marker of the DFC, fibrillarin (FBL) was next employed. Although the pattern of titin and FBL immunofluorescence appeared virtually identical using standard epifluorescence microscopy, laser scanning confocal microscopy (LSCM) demonstrates that the two antibodies recognize partially distinct domains (arrows and arrowheads in Fig. 3F–H and Movie 1). Overall, LSCM for titin reveals a spherical reticulate pattern that is closely intertwined with FBL and demarcated by DAPI+ NORs. (Fig 3. F–L, Movies 1 and 2). Within the DFC, FBL and other rRNA processing factors cluster into ring-like structures around the fibrillar center (FC) (21, 22). These DFC/FC modules are thought to serve as reaction chambers for individual ribosome maturation (21, 23). Figure 3L (asterisk) and Movie 2 show that within the DFC, titin localizes to these clustered rings. Also consistent with titin’s localization to the DFC, Figures 3M–P show that titin immunostaining is surrounded by NPM1, a marker of the granular component that envelopes the DFC. In final support of nucleolar localization, the online nucleolar localization sequence detector tool, NoD, identifies 20 nucleolar localization sequences in human titin (Fig. E2) (24).

### Titin is present in multiple neuronal cell types and Schwann cells in the dorsal root ganglia, sympathetic ganglia, and spinal cord

**Figure E3.**
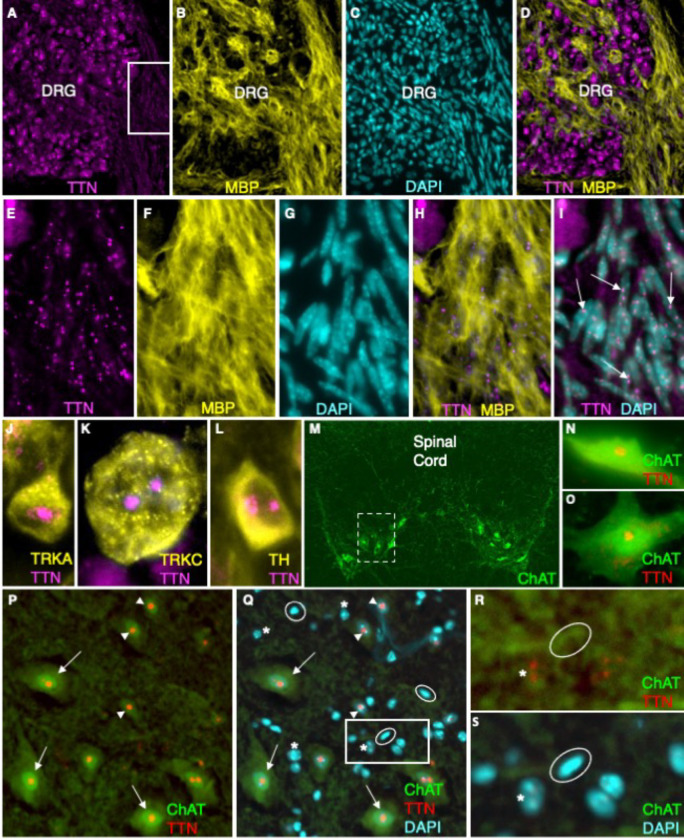
Titin is present in multiple neuronal cell types and Schwann cells in the DRG, sympathetic ganglia, and spinal cord. (A-L) E17.5-E18.5. (A-I) Titin is present in both neurons and Schwann cells (MBP-positive) in the DRG. (E-I) An enlargement of the boxed region in A. (E, H, I) Titin (magenta) was artificially brightened to enhance its visualization. (J-L) IHC using subtype specific antibodies shows that both small-diameter, TRKA+ nociceptors (J), and large-diameter, TRKC+ proprioceptors (K) are titin+. (L) Nucleolar titin is also present in tyrosine hydroxylase (TH)+ neurons in the autonomic sympathetic ganglia. (M-S) Spinal cords from *Chat-EGFP* mice in which GFP is selectively expressed in cholinergic neurons. (N,O) Titin is present in both embryonic (E17.5) (N) and adult (O, P, Q) (6 weeks) cholinergic motor neurons. (P,Q) The same spinal cord section (with and without DAPI) and corresponding to a region of the spinal cord designated by the dashed box in (M). (R,S) An enlargement of the boxed region shown in (Q). The amount of titin is greater in the cholinergic EGFP-positive alpha and gamma motor neurons (arrows and arrowheads in (P,Q), respectively) compared to EGFP-negative cells with more diffuse DAPI staining (interneurons) (asterisks in Q-S) and EGFP-negative glia (cells with triangular or oblong nuclei that stain densely with DAPI) (circled in Q-S) where titin levels are minimal or absent. DRG, dorsal root ganglia; MBP, myelin binding protein; ChAT, choline acetyltransferase; TRKA, receptor tyrosine kinase A; TRKC, receptor tyrosine kinase C. Bar = 45 um in (A-D); 15 um in E-I; 6 um in (J-L); 225 um in (M); 12 um in (N,O); 25 um in (P,Q); 60 um in (R,S).

Within the peripheral nervous system, titin is present in the nucleolus of both neurons and Schwann cells (Fig. E3). However, the amount of titin in Schwann cells is dramatically less than in their neuronal counterparts (Fig. E3A–I). IHC using subtype specific antibodies shows that both small-diameter and large-diameter sensory neurons are titin+ (Fig. E3J,K). Robust immunostaining is also present in nucleoli of neurons in the autonomic sympathetic ganglia (Fig. E3L). Within the central nervous system, IHC was performed on spinal cord sections from *ChAT-EGFP* mice in which EGFP is selectively expressed in cholinergic neurons (Fig. E3M) (25). Nucleolar titin immunostaining was observed in all cholinergic neurons, with large alpha motor neurons in the ventral horn exhibiting the most robust fluorescence and the largest puncti at both embryonic and adult time points (Fig. 2B, E3N–Q). Titin was also observed in smaller GFP+ gamma motor neurons (arrowheads in Fig. E3P,Q). Notably, titin levels are significantly more robust in the cholinergic, EGFP+ subpopulation within the spinal cord compared to EGFP-negative cells with more diffuse DAPI staining (likely interneurons) (asterisks in Fig. E3Q–S) and EGFP-negative glia (cells with triangular or oblong nuclei that stain densely with DAPI (26, 27)) (circled in Fig. SEQ-S) where titin levels are minimal or absent. These findings align with the relative expression of *Ttn* observed via RT-qPCR; while titin protein is present in most sensory neuron subtypes in the DRG and in Schwann cells, corresponding to its relatively abundant transcript, titin expression is more restricted in the spinal cord, with cholinergic motor neurons exhibiting the highest levels. Since we did not isolate motor neurons from the other cell types present in the cord, it follows that its expression level within the spinal cord as a whole would be quite low.

### Titin is present in multiple neuronal cell types in the brain

**Figure E4.**
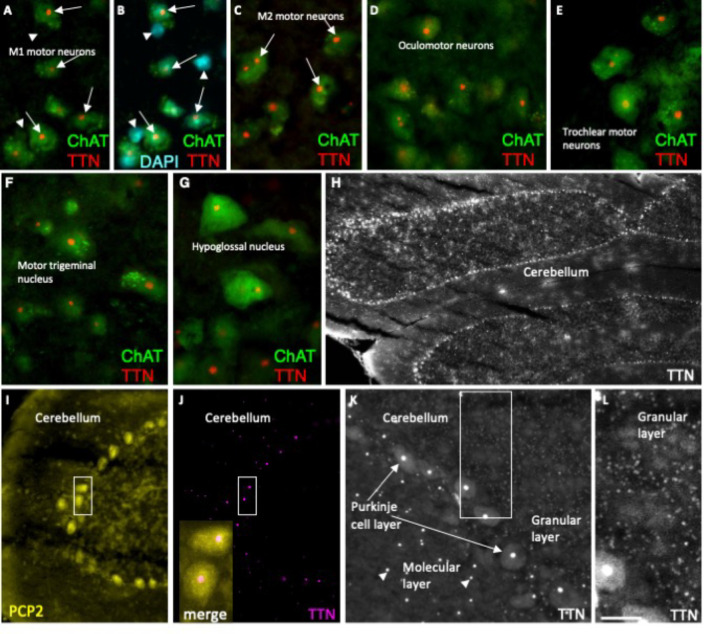
Titin is present in multiple neuronal cell types in the brain. (A-L) Coronal sections of the adult (6 weeks) mouse brain. (A-C) Within the cortex, titin is observed in the nucleolus of M1 and M2 motor neurons (arrows), but not observed in non-cholinergic, EGFP-negative cells (arrowheads in B). (D-G) Neurons of the oculomotor, trochlear, trigeminal, and hypoglossal nuclei exhibit nucleolar titin. (H-L) Titin expression is widespread in neurons of the cerebellum with the amount of titin corresponding to cell size; large Purkinje neurons in I-K exhibit the highest titin levels per cell, medium-sized neurons in the molecular layer exhibit intermediate levels, and small neurons of the granular layer show minimal titin. Inset in J is an enlargement and merge of the boxed region shown in I and J. L shows an enlargement of the boxed region in K. ChAT, choline acetyltransferase; PCP2, Purkinje cell protein 2. Bar = 25 um in (A-G), 150 um in (H), 70 um in (I,J), 35 um in (K), 18 un in (L).

Within the adult cortex, titin is observed in the nucleolus of M1 and M2 motor neurons (arrows in Fig. E4A–C), but not observed in non-cholinergic, EGFP-negative cells (arrowheads in Fig. E4B). Neurons of the oculomotor, trochlear, trigeminal, and hypoglossal nuclei all exhibit nucleolar titin (Fig. E4D–G). Finally, titin expression is widespread in neurons of the cerebellum with its amount corresponding to cell size; large Purkinje neurons exhibit the highest titin levels per cell, neurons in the molecular layer exhibit intermediate levels, and the small, tightly packed neurons of the granular layer show minimal amounts of titin (Fig. E4H–L).

### Titin may participate in LLPS

**Figure E5.**
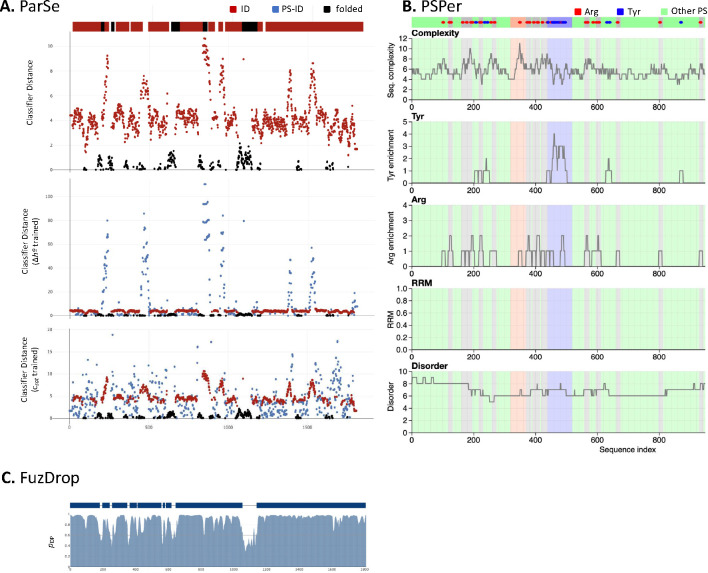
Three phase-prediction algorithms that generate sequence-dependent predictions of phase-separation behavior in the PEVK domain of titin. (A) Of the three variations of ParSe, the Δ*h*°-trained and *c*_sat_-trained algorithms both predict dispersed phase-separation potential across the PEVK sequence (middle and bottom plots). Residues and domains are identified as phase-separating intrinsically-disordered (PS-IDP, blue), non-phase-separating intrinsically-disordered (ID, red) and or folded (F, black). The domain topology diagram above the plots represents the aggregate analysis by the baseline algorithm (top plot). (B) PSPer combines sequence analysis of disorder, complexity, arginine, and tyrosine content to yield an overall PSP score, reported in Table 1. (C) FuzDrop also predicts distributed potential for phase separation (*p*_DP_, residue-based droplet-promoting probability). Contiguous regions of high potential are identified as potentially phase-separating domains, represented in the topology diagram in dark blue.

Nucleolar scaffolding proteins, including nucleophosmin, undergo heterotypic interactions with other nucleolar components, including proteins that harbor R-motifs and intrinsically disordered regions, to drive liquid-liquid phase separation (LLPS) and formation of the liquid-like condensate of the nucleolus (28, 29). LLPS may also direct pre-ribosomal particle assembly within the nucleolus and exit from the nucleolus as part of the ribosome biogenesis process (28). Titin houses hundreds of intrinsically disordered regions as well as numerous R-motifs within its PEVK region, suggesting that titin may participate in LLPS (30). To explore this possibility, we evaluated the PEVK sequence of human titin using six web-based prediction algorithms of phase separation potential (catGRANULE, FuzDrop, ParSe, PScore, PSPer, and PSPredictor). Four algorithms predicted that the PEVK sequence could exhibit liquid-liquid phase separation (Table 1). The positive predictions spanned the entire PEVK region, with no clear sub-region identified as being enriched in phase-separation potential (Fig. E5).

### Titin levels are altered in SOD1^G93A^ model mice

**Figure E6.**
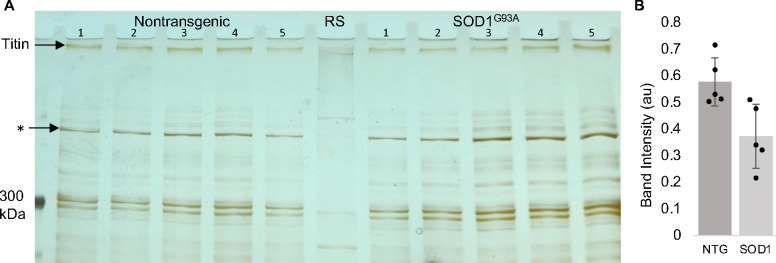
Titin levels are diminished in the cortex of SOD1^G93A^ mice. (A) PAGE analysis shows that titin protein levels in the cortex of SOD1^G93A^ mice (lanes on the right half of the gel) are slightly reduced compared to nontransgenic (NTG) controls (lanes on the left half of the gel). Asterisk indicates the band used for normalization during densitometry analysis. (B) Plot of titin densitometry data. p = 0.016. RS, rabbit soleus.

**Figure E7.**
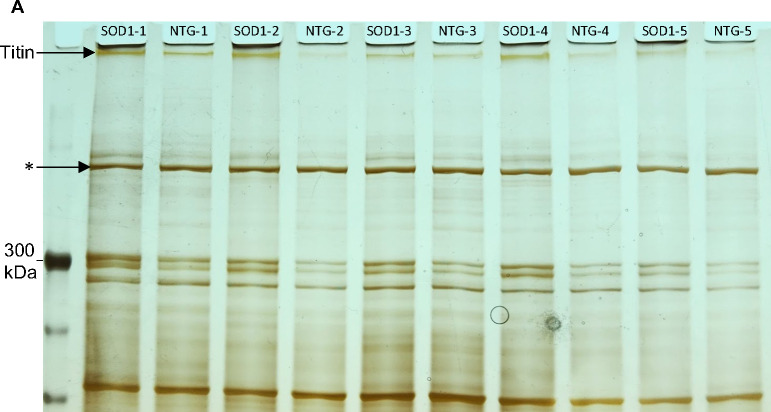
Titin levels are elevated in the spinal cords of SOD1^G93A^ mice. (A) PAGE analysis shows that titin protein levels in the spinal cords of SOD1^G93A^ mice (SOD1) are elevated compared to nontransgenic (NTG) age-matched controls. Asterisk indicates the band used for normalization. See Fig. 4 for titin densitometry analysis.

**Figure 4.**
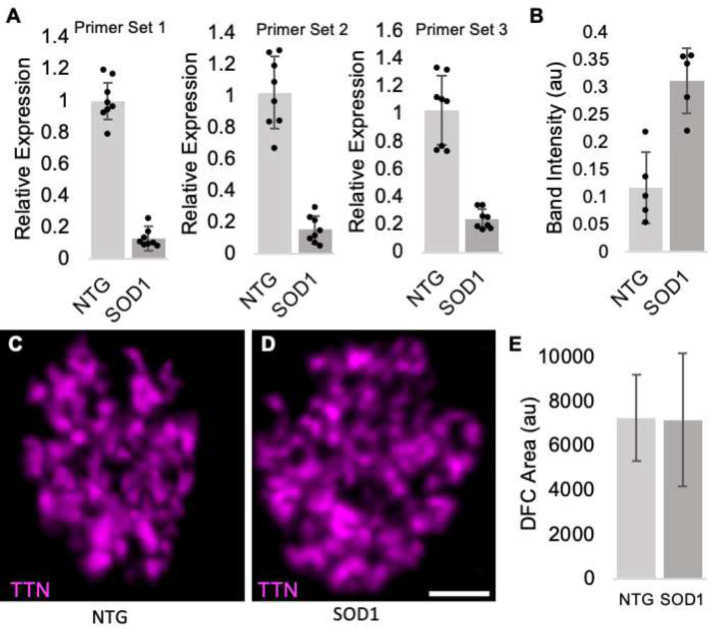
Titin levels are altered in SOD1^G93A^ mice. (A) RT-qPCR using three different sets of primers shows that *Ttn* mRNA levels are diminished by approximately eight-fold in spinal cords of SOD1^G93A^ mice compared to their NTG counterparts (p < 0.0001 for each). (B) PAGE and densitometry analysis indicate that titin protein levels are elevated in the spinal cords of SOD1^G93A^ mice (p = 0.001) (see also Fig E7). (C,D) LSCM shows that titin patterning in the DFC of alpha motor neurons in SOD1^G93A^ mice is comparable to NTG controls. (E) The average size of the DFC in spinal cord alpha motor neurons of SOD1^G93A^ mice is also normal (p = 0.86; n = 3 spinal cords and 40 cells per genotype). au, arbitrary units.

Diminished levels of titin protein in the brains of 12-week SOD1^G93A^ mice were previously reported via mass spectrometry (31). Similarly, we find slightly reduced levels of titin in the cortex of SOD1^G93A^ mice at the end stage of disease (P120) (Fig. E6). Since lower motor neurons are the most damaged in SOD1^G93A^ mice (32), we also examined both transcript and protein titin levels in spinal cords of SOD1^G93A^ mice and nontransgenic (NTG) controls. As shown in Figure 4, although mRNA levels of *Ttn* are reduced in the mutant by approximately 8-fold, *Ttn* protein levels are elevated (Fig. 4B, Fig. E7). Using IHC and LSCM we also observed titin’s nucleolar pattern in the remaining alpha motor neurons present in SOD1^G93A^ spinal cords. As shown in Figure 4, the majority of these neurons exhibit a normal nucleolar titin pattern with an intensity comparable to their NTG counterparts (Fig. 4C,D). In addition, although the area of the DFC is more variable in the spinal cords of SOD1^G93A^ mice, the average area is the same as NTG controls (Fig. 4E).

## Discussion

Here we demonstrate that titin resides in the DFC of the neuron nucleolus. Although titin has been detected in previous neuronal proteome and transcriptome studies, its robust presence in the nucleolus of numerous neuronal subtypes is a surprising finding. At 3600 kDa, titin’s size may have interfered with its detection previously.

Within the sarcomere, titin adopts a spring-like structure. Although our PAGE analyses of neuronal titin indicate that at least one isoform represents a molecule of similar size, neuronal titin does not appear to adopt the same three-dimensional conformation. Titin’s size alone, as well as our analyses demonstrating that it occupies much of the DFC area and spatially overlaps with that of fibrillarin, suggest that titin may serve as a scaffold for formation of the DFC and localization of rRNA modification enzymes. Our computational analyses indicating that titin’s intrinsically disordered region has the potential to phase separate are also consistent with a scaffolding function, although additional studies that directly investigate LLPS are needed. Titin’s localization to the DFC suggests that in general, titin may contribute to ribosome production and translational homeostasis, two overlapping processes with strong ties to neurodegenerative disease. Intriguingly, polymorphisms in the *TTN* gene and reduced *TTN* expression are in fact associated with ALS and an increased severity of disease (13). Although this increase in severity may be due to decreased skeletal muscle function (13), it is also possible that attenuation of titin’s function in the neuron nucleolus is the true pathophysiologic driver. Indeed, studies demonstrating that nucleolar stress is an early feature of both familial and sporadic forms of ALS indicate that nucleolar dysfunction may in fact represent a common pathogenic nexus of disease (12).

The majority of ALS cases (~90%) are sporadic, with no family history (33, 34). The remaining 10% are inherited and associated with a list of more than 40 genes. Mutations in just four of these genes account for ~70% of familial cases (*C9orf72, SOD1, TARDBP and FUS*) (35, 36). All ALS cases are characterized by insoluble cytosolic protein aggregates in neurons (37, 38). Although aggregated TDP-43, the protein encoded by *TARDBP*, is not present in *SOD1*- or *FUS*-related disease, it is a major component of disease aggregates in nearly all sporadic ALS cases (97%) and in most SOD-1-negative familial cases (39, 40). Interestingly, studies have shown that *TTN* RNA is present in TDP-43 amyloid aggregates that form in muscle during regeneration. These aggregates are structurally similar to the aggregates found in neurons of patients with ALS (41). Given the sheer size of titin’s transcript, as well as a plethora of TDP-43 binding sites (41), it is tempting to speculate that titin’s transcript might play a role in nucleating TDP-43 and in triggering the formation of aggregates (42). Although the inclusions in *SOD1*-related ALS are TDP-43-negative, it is possible that titin’s message may still contribute to the formation of neuronal aggregates that are known to be rich in RNAs. The potential sequestering of *Ttn* mRNA in aggregates could also explain why *Ttn* transcript levels are reduced in SOD1^G93A^ mice.

Containing more than 200 immunoglobulin domains, motifs prone to aggregation (43), titin protein may also contribute to aggregates in ALS. *In vitro* studies demonstrating the aggregation of specific titin immunoglobulins lend support to this hypothesis (44). Here we show that SOD1^G93A^ mice have elevated levels of titin protein in the spinal cord. Although cytoplasmic titin aggregates were not detected in motor neurons of SOD1^G93A^ mice via IHC, it is possible that antibody 27867-1-AP does not recognize misfolded and/or aggregated titin. This could explain how titin protein levels are elevated in SOD1^G93A^ mice in the context of diminished messenger RNA. Elevated titin levels could also result from a dysfunctional ubiquitin proteasome system in SOD1^G93A^ mice (45). Our data demonstrating that titin protein levels are not depleted in the spinal cords of SOD1^G93A^ mice, and that titin patterning appears normal suggests that titin’s potential contribution to ALS results from a role other than a diminishment of function.

Here we demonstrate that titin is broadly expressed in neurons of both the peripheral and central nervous systems. Although ALS primarily impacts central nervous system components, multiple studies have shown that peripheral sensory and autonomic neurons are affected as well (46–48). Thus, our demonstration of titin in peripheral DRG and sympathetic neurons, in addition to central components, is still consistent with a potential role for titin in ALS. Indeed, understanding why alpha motor neurons are most vulnerable to ALS-associated mutations while other neuronal subtypes are less impacted, remains a significant challenge in the field. One obvious difference is size; alpha motor neurons are the largest neurons in the spinal cord. Studies have shown that the number of DFC/FC modules present in the nucleolus is cell type dependent (reviewed in Lafontaine *et al*.) (21). Given the large size of alpha motor neurons, it follows that they likely have a uniquely large ribosomal burden and correspondingly large number of DFC/FC modules. Hence, the large difference in the amount of titin observed in neurons compared to other cell types including Schwann cells may simply reflect the much larger ribosomal burden carried by neurons. This ribosomal load may make neurons, particularly large neurons like alpha motor neurons, particularly sensitive to cellular insults that compromise ribosome production.

The unveiling of titin, a colossal protein encoded by an equally colossal transcript, as a nucleolar resident stands to transform our understanding of the molecular functioning and formation of this organelle. Moreover, given the emerging central role that nucleolar dysfunction plays in neurological disease, a more comprehensive understanding of nucleolar dynamics, including titin’s contribution, will almost certainly reveal new avenues for the development of neuroprotective therapies.

## Materials and Methods

### Immunohistochemistry and imaging.

Except Figs. 4C,D, spinal cords were isolated via hydraulic extrusion, the lumbar enlargement isolated, fixed for 2.25 hours in 4% paraformaldehyde, and worked through a sucrose gradient (15% sucrose in PBS until the tissue sinks, followed by 30% sucrose until the tissue sinks). Spinal cords were then frozen and embedded in OCT in a dry ice ETOH bath and sectioned at 16 uM. IHC was performed as previously described (8) with the addition of an antigen retrieval step [10 min at 95° C in citric acid buffer (10mM citric acid, 0.05% Tween 20, pH 6.0)] for titin staining. For Figs. 4C,D, 16 uM sections were cut directly from frozen lumbar enlargement regions of the spinal cord, incubated in 4% paraformaldyhyde X 20 minutes, rinsed 3X in PBS, followed by IHC as described above. Brain nuclei were identified using *ChAT-EGFP* mice and *The Mouse Brain in Stereotaxic Coordinates* (49). Primary antibodies included the following: anti-Titin (Proteintech, 27867-1-AP, 1:500), anti-Fibrillarin (Santa Cruz Biologicals, sc-374022, 1: 50), anti-Tuj1 (Biolegends, 801202, 1:1000), anti-NPM1–594 (Proteintech, CL594–60096, 1:100), Anti-TrkA (R&D Systems, AF1056-SP, 5 ug/ml), Anti-TrkC (R&D Systems, AF1404, 1:500), anti-TH (Santa Cruz Biotechnology, sc-25269, 1:200), Anti-GFP (Abcam, ab13970, 1:1000), Anti-PCP2 (Proteintech, 13774-1-AP, 1:100). Alexa Fluor secondary antibodies (Thermo Fisher) were used at a dilution of 1:2000. Except for Figs. 3F–L, and 4C,D, microscopy images were captured using a Nikon TE200 inverted microscope with a QImaging QICAM 12 bit Mono Fast 1394 Cooled camera and SPOT software.

### Leica Stellaris 8 Confocal Imaging.

High-resolution images shown in Figs. 3F–L and 4C,D were acquired with a Leica Stellaris 8 Confocal Scanning Laser Microscope (CSLM) using the LIGHTNING 3D Deconvolution mode. Excitation and emission settings were tuned specifically for each fluorophore or groups of fluorophores to reduce and eliminate crosstalk. Fluorescent labels and were excited with either a diode 405 nm laser (DAPI em:425–494nm), or a white light laser: Alexa Fluor 488 (499/504–584nm) for titin , and Alexa Fluor 568 (579/584–728) for fibrillarin. Multiple channels were scanned sequentially, and all channels were line averaged 3 times. Photons were detected with a combination of HyD S and HyD X detectors. A HC PL APO 63x/1.40 Oil CS2 was used for all CSLM image collection, the pinhole was set at 1.00 AU and a confocal zoom was applied to further increase magnification. Z-stack images from selected regions of interest were captured at Nyquist lateral and axial resolutions and adaptive deconvolution was calculated for a 1.52 refractive index. Deconvolved z-stacks were projected and processed in Leica LASX software (Version 4.5.0.25531).

### RT-qPCR.

Figure 1B: Reverse transcriptase quantitative PCR (RT-qPCR) for titin was performed using the Viia-7 qPCR System by Applied Biosystems and TaqMan Gene Expression Assay Mm00621005 (ThermoFisher #4331182). RNA was extracted (Qiagen RNeasy) and cDNA synthesized (Applied Biosystems RNA-to-cDNA). Each reaction was performed in triplicate. Bars represent SD calculated from three Delta Delta Ct Expression values for each tissue type. Figure 4A: RNA was extracted from spinal cords of SOD1^G93A^ transgenic mice (JAX #002726) and corresponding non-transgenic (NTG) littermate controls (n=8 for each group) (P120+/−7) with RNeasy^®^ Plus Mini Kit (Qiagen) in combination with the RNase-Free DNase set (Qiagen) following the manufacturer’s instruction. A total of 0.5 μg RNA was converted into cDNA using qScript^™^ cDNA kit from QuantaBio according to the manufacturer’s protocol. qPCR was performed by Applied Biosystems^™^ StepOnePlus^™^ Real-Time PCR System. Each reaction in a total volume of 20 μl contained 50 ng cDNA, 10 μl TaqMan^™^ Fast Advanced Master Mix (ThermoFisher), and 1 μl Taqman probe targeting *Titin* (Mm00621005_m1) or the housekeeping *Gapdh* (Mm99999915_g1). Expression of *Titin* mRNA was also confirmed by SYBR green method with 2 additional sets of primers, namely Ttn_70K (Forward: 5’-GAGTACTTCTTCCGGGTCTTTG-3’, and Reverse: 5’-GTTCTAGCTGGTCCTTGATGAG-3’) and Ttn_80K (Forward: 5’-CACCTGGAGGAAAGATGAGAAG-3’, and Reverse: 5’-TGCGAGTGACTTGAGGAATAAC-3’). Similarly, each reaction contained 50 ng cDNA, 10 μl Power SYBR^™^ Green PCR Master Mix (ThermoFisher) and 200 nm primer synthesized from IDTDNA. For the SYBR green method, *Gapdh* (Forward: 5’-CATCACTGCCACCCAGAAGACTG-3’, and Reverse: 5’-ATGCCAGTGAGCTTCCCGTTCAG-3’) was also amplified simultaneously as endogenous control for normalization. The relative changes in *Ttn* expression were normalized against *Gapdh* and were analyzed by 2^(−ΔΔCt) method as described previously (50).

### PAGE.

Lysates were prepped using N-PER Neuronal Protein Extraction Reagent (Thermo Fisher #87792) according to the manufacturer’s instructions or a Nuclear Extraction Kit (Abcam #ab113474), or in the case of muscle, a previously published protocol was used (51). Protease inhibitor (Thermo Fisher Scientific) was added to each sample before homogenization. Protein concentrations of all samples were determined by using a bicinchroninic acid (BCA) or Bradford protein assay (Thermo Fisher Scientific). Equal amounts of protein were incubated with XT sample buffer and reducing agent for 35–40 min at 37 C and run on 3–8% tris-acetate gels from either Fisher Scientific (#EA0375) or Bio-Rad (#3550129). Gels were run for a total of 18–19 hours overnight at 4 C beginning with a voltage of 2mV for 40min and then increasing to 5mV. At the 2.5 hr mark, buffer was renewed and voltage was increased to 10mV for remaining run time. Titin protein bands were visualized in samples by staining with Pierce Silver Stain (Thermo Fisher Scientific) and quantified using NIH ImageJ software and analyzed via unpaired t-test.

### Mass Spectroscopy.

Since silver staining is not compatible with MS and Coomassie is not sensitive enough to visualize low abundance neuronal titin, the following approach was used: duplicate lysates were run on each half of a gel and one half was stained with Pierce Silver Stain for visualizing the location of bands, and the other half with Coomassie for MS (Fig. S9). In our hands, when mouse gastrocnemius muscle is prepped as described in Warren *et al*., 2003 (51), a majority of titin is present as break down products. These smaller products enter the gel more easily and are abundant enough to be visualized with Coomassie, providing an additional visual marker for estimating the location of the larger neuronal titin bands of interest that were excised (Fig. S8). Samples were processed by MS Bioworks https://www.msbioworks.com/. For sample preparation, in-gel digestion with trypsin was performed using a DigestPro robot (CEM) as follows: Samples were washed with 25mM ammonium bicarbonate followed by acetonitrile, reduced with 10mM dithiothreitol at 60°C followed by alkylation with 50mM iodoacetamide at room temperature, digested with sequencing grade trypsin (Promega) at 37°C for 4h, quenched with formic acid, and the supernatant was analyzed directly without further processing. For mass spectrometry, half of each digested sample was analyzed by nano LC-MS/MS with a Waters NanoAcquity HPLC system interfaced to a ThermoFisher Fusion Lumos mass spectrometer. Peptides were loaded on a trapping column and eluted over a 75μm analytical column at 350nL/min; both columns were packed with Luna C18 resin (Phenomenex). The mass spectrometer was operated in data-dependent mode, with the Orbitrap operating at 60,000 FWHM and 15,000 FWHM for MS and MS/MS respectively. The instrument was run with a 3s cycle for MS and MS/MS. 30 Minutes of run timeData were searched using a local copy of Mascot (Matrix Science) with the following parameters: Enzyme: Trypsin/P Database: A: SwissProt Mouse (concatenated forward and reverse plus common contaminants) B: UniProt Rabbit (concatenated forward and reverse plus common contaminants) Fixed modification: Carbamidomethyl (C) Variable modifications: Oxidation (M), Acetyl (N-term), Pyro-Glu (N-term Q), Deamidation (N/Q) Mass values: Monoisotopic Peptide Mass Tolerance: 10 ppm Fragment Mass Tolerance: 0.02 Da Max Missed Cleavages: 2 Mascot DAT files were parsed into Scaffold (Proteome Software) for validation, filtering and to create a non-redundant list per sample. Data were filtered using at 1% protein and peptide FDR and requiring at least two unique peptides per protein.

### Mice.

Chat-EGFP and SOD1^G93A^ mice were purchased from the Jackson Laboratory (stock # 007902 and 002726, respectively). All experiments were performed according to the National Institutes of Health Guide for Care and Use of Laboratory Animals, and protocols were approved by the Montana State University and Northwestern University Institutional Animal Care and Use Committees.

## Extended Data

**Figure E8.**
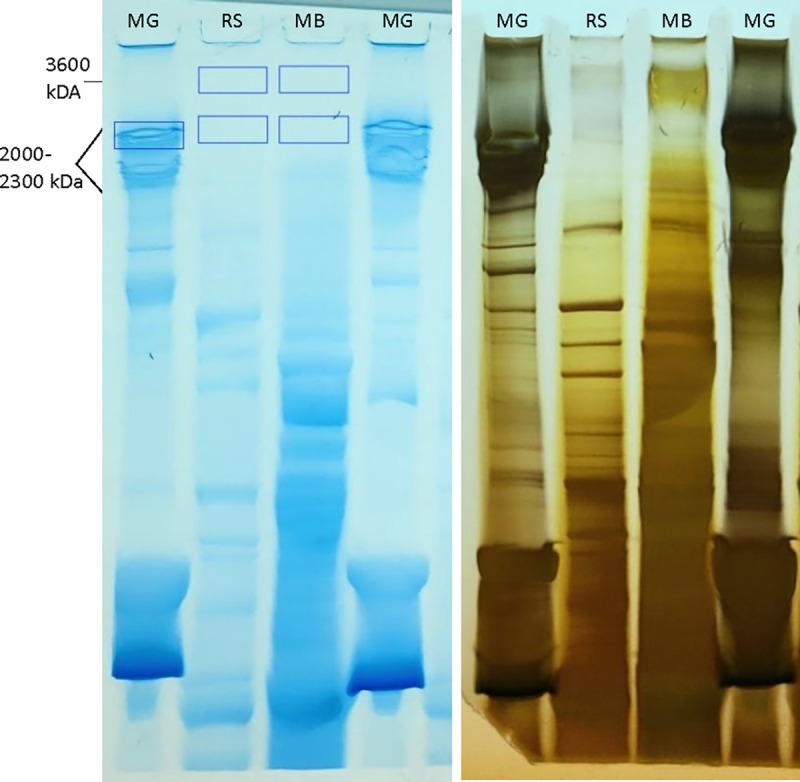
Gels used for mass spectrometry. Since silver staining is not compatible with MS and Coomassie is not sensitive enough to visualize low abundance neuronal titin, the following approach was used: duplicate lysates were run on each half of a gel and one half was stained with Pierce Silver Stain for visualizing the location of bands, and the other half with Coomassie for MS. In our hands, when mouse gastrocnemius muscle is prepped as described in Warren *et al*., 2003 (51), a majority of titin is present as break down products. These smaller products enter the gel more easily and are abundant enough to be visualized with Coomassie, providing an additional visual marker for estimating the location of the larger neuronal titin bands of interest. The blue boxes in the Coomassie stained gel show the regions excised for MS analysis. MG, mouse gastrocnemius muscle; RS, rabbit soleus; MB, mouse brain.

## Figures and Tables

**Table 1.** Several LLPS prediction algorithms identify a potential phase-separation behavior for the titin PEVK sequence.

Algorithm	Score	LLPS?

**catGRANULE** ^1^	0.24	n
**FuzDrop** ^2^	0.9995	**Y**
**ParSe 2.0** ^3^		
baseline	0	n
∆*h*º	9819.3	**Y**
c_sat_	2967.4	**Y**
**PScore** ^1^	2.82	n
**PSPer** ^1^	0.605	**Y**
**PSPredictor** ^1^	0.9808	**Y**

Calculated metrics:

1 score;

2 p(LLPS);

3 sum of classifier distance.

## References

[1] Di Cola E, Waigh TA, Trinick J, Tskhovrebova L, Houmeida A, Pyckhout-Hintzen W, Dewhurst C. Persistence length of titin from rabbit skeletal muscles measured with scattering and microrheology techniques. Biophys J. 2005;88(6):4095–106. Epub 2005/03/29. doi: 10.1529/biophysj.104.054908. 15792980 PMC1305640

[2] Minajeva A, Kulke M, Fernandez JM, Linke WA. Unfolding of titin domains explains the viscoelastic behavior of skeletal myofibrils. Biophys J. 2001;80(3):1442–51. Epub 2001/02/27. doi: 10.1016/S0006-3495(01)76116-4. 11222304 PMC1301335

[3] Aladesuyi Arogundade O, Stauffer JE, Saberi S, Diaz-Garcia S, Malik S, Basilim H, Rodriguez MJ, Ohkubo T, Ravits J. Antisense RNA foci are associated with nucleoli and TDP-43 mislocalization in C9orf72-ALS/FTD: a quantitative study. Acta Neuropathol. 2019;137(3):527–30. Epub 2019/01/23. doi: 10.1007/s00401-018-01955-0. 30666413 PMC6397670

[4] Lehmkuhl EM, Zarnescu DC. Lost in Translation: Evidence for Protein Synthesis Deficits in ALS/FTD and Related Neurodegenerative Diseases. Adv Neurobiol. 2018;20:283–301. Epub 2018/06/20. doi: 10.1007/978-3-319-89689-2_11. 29916024 PMC6530776

[5] Parlato R, Kreiner G. Nucleolar activity in neurodegenerative diseases: a missing piece of the puzzle? J Mol Med (Berl). 2013;91(5):541–7. Epub 2012/11/28. doi: 10.1007/s00109-012-0981-1. 23179684 PMC3644402

[6] Rudolph F, Huttemeister J, da Silva Lopes K, Juttner R, Yu L, Bergmann N, Friedrich D, Preibisch S, Wagner E, Lehnart SE, Gregorio CC, Gotthardt M. Resolving titin’s lifecycle and the spatial organization of protein turnover in mouse cardiomyocytes. Proc Natl Acad Sci U S A. 2019;116(50):25126–36. Epub 2019/11/24. doi: 10.1073/pnas.1904385116. 31757849 PMC6911189

[7] Machado C, Andrew DJ. D-Titin: a giant protein with dual roles in chromosomes and muscles. J Cell Biol. 2000;151(3):639–52. Epub 2000/11/04. doi: 10.1083/jcb.151.3.639. 11062264 PMC2185597

[8] Goffena J, Lefcort F, Zhang Y, Lehrmann E, Chaverra M, Felig J, Walters J, Buksch R, Becker KG, George L. Elongator and codon bias regulate protein levels in mammalian peripheral neurons. Nat Commun. 2018;9(1):889. doi: 10.1038/s41467-018-03221-z. 29497044 PMC5832791

[9] Jovicic A, Mertens J, Boeynaems S, Bogaert E, Chai N, Yamada SB, Paul JW, 3rd, Sun S, Herdy JR, Bieri G, Kramer NJ, Gage FH, Van Den Bosch L, Robberecht W, Gitler AD. Modifiers of C9orf72 dipeptide repeat toxicity connect nucleocytoplasmic transport defects to FTD/ALS. Nat Neurosci. 2015;18(9):1226–9. Epub 2015/08/27. doi: 10.1038/nn.4085. 26308983 PMC4552077

[10] Kwon I, Xiang S, Kato M, Wu L, Theodoropoulos P, Wang T, Kim J, Yun J, Xie Y, McKnight SL. Poly-dipeptides encoded by the C9orf72 repeats bind nucleoli, impede RNA biogenesis, and kill cells. Science. 2014;345(6201):1139–45. Epub 2014/08/02. doi: 10.1126/science.1254917. 25081482 PMC4459787

[11] Haeusler AR, Donnelly CJ, Periz G, Simko EA, Shaw PG, Kim MS, Maragakis NJ, Troncoso JC, Pandey A, Sattler R, Rothstein JD, Wang J. C9orf72 nucleotide repeat structures initiate molecular cascades of disease. Nature. 2014;507(7491):195–200. Epub 2014/03/07. doi: 10.1038/nature13124. 24598541 PMC4046618

[12] Aladesuyi Arogundade O, Nguyen S, Leung R, Wainio D, Rodriguez M, Ravits J. Nucleolar stress in C9orf72 and sporadic ALS spinal motor neurons precedes TDP-43 mislocalization. Acta Neuropathol Commun. 2021;9(1):26. Epub 2021/02/17. doi: 10.1186/s40478-021-01125-6. 33588953 PMC7885352

[13] Watanabe H, Atsuta N, Hirakawa A, Nakamura R, Nakatochi M, Ishigaki S, Iida A, Ikegawa S, Kubo M, Yokoi D, Watanabe H, Ito M, Katsuno M, Izumi Y, Morita M, Kanai K, Taniguchi A, Aiba I, Abe K, Mizoguchi K, Oda M, Kano O, Okamoto K, Kuwabara S, Hasegawa K, Imai T, Kawata A, Aoki M, Tsuji S, Nakashima K, Kaji R, Sobue G. A rapid functional decline type of amyotrophic lateral sclerosis is linked to low expression of TTN. Journal of neurology, neurosurgery, and psychiatry. 2016;87(8):851–8. Epub 2016/01/10. doi: 10.1136/jnnp-2015-311541. 26746183

[14] Blum JA, Klemm S, Shadrach JL, Guttenplan KA, Nakayama L, Kathiria A, Hoang PT, Gautier O, Kaltschmidt JA, Greenleaf WJ, Gitler AD. Single-cell transcriptomic analysis of the adult mouse spinal cord reveals molecular diversity of autonomic and skeletal motor neurons. Nat Neurosci. 2021;24(4):572–83. Epub 2021/02/17. doi: 10.1038/s41593-020-00795-0. 33589834 PMC8016743

[15] Alkaslasi MR, Piccus ZE, Hareendran S, Silberberg H, Chen L, Zhang Y, Petros TJ, Le Pichon CE. Single nucleus RNA-sequencing defines unexpected diversity of cholinergic neuron types in the adult mouse spinal cord. Nat Commun. 2021;12(1):2471. Epub 2021/05/02. doi: 10.1038/s41467-021-22691-2. 33931636 PMC8087807

[16] Human Protein Atlas. Available from: https://www.proteinatlas.org/humanproteome/brain.

[17] Hasan M, Min H, Rahaman KA, Muresan AR, Kim H, Han D, Kwon OS. Quantitative Proteome Analysis of Brain Subregions and Spinal Cord from Experimental Autoimmune Encephalomyelitis Mice by TMT-Based Mass Spectrometry. Proteomics. 2019;19(5):e1800355. Epub 2019/02/07. doi: 10.1002/pmic.201800355. 30724464

[18] Prado LG, Makarenko I, Andresen C, Kruger M, Opitz CA, Linke WA. Isoform diversity of giant proteins in relation to passive and active contractile properties of rabbit skeletal muscles. J Gen Physiol. 2005;126(5):461–80. Epub 2005/10/19. doi: 10.1085/jgp.200509364. 16230467 PMC2266601

[19] Wang SM, Greaser ML. Immunocytochemical studies using a monoclonal antibody to bovine cardiac titin on intact and extracted myofibrils. J Muscle Res Cell Motil. 1985;6(3):293–312. Epub 1985/06/01. doi: 10.1007/BF00713171. 3905857

[20] Pontvianne F, Carpentier MC, Durut N, Pavlistova V, Jaske K, Schorova S, Parrinello H, Rohmer M, Pikaard CS, Fojtova M, Fajkus J, Saez-Vasquez J. Identification of Nucleolus-Associated Chromatin Domains Reveals a Role for the Nucleolus in 3 D Organization of the A. thaliana Genome. Cell reports. 2016;16(6):1574–87. Epub 2016/08/02. doi: 10.1016/j.celrep.2016.07.016. PMC5279810 27477271

[21] Lafontaine D, Riback JA, Bascetin R, Brangwynne CP. The nucleolus as a multiphase liquid condensate. Nat Rev Mol Cell Biol. 2021;22:165–82. Epub 9/1/2020. 32873929 10.1038/s41580-020-0272-6

[22] Yao RW, Xu G, Wang Y, Shan L, Luan PF, Wang Y, Wu M, Yang LZ, Xing YH, Yang L, Chen LL. Nascent Pre-rRNA Sorting via Phase Separation Drives the Assembly of Dense Fibrillar Components in the Human Nucleolus. Molecular cell. 2019;76(5):767–83 e11. Epub 2019/09/22. doi: 10.1016/j.molcel.2019.08.014. 31540874

[23] Scheer U, Hugle B, Hazan R, Rose KM. Drug-induced dispersal of transcribed rRNA genes and transcriptional products: immunolocalization and silver staining of different nucleolar components in rat cells treated with 5,6-dichloro-beta-D-ribofuranosylbenzimidazole. J Cell Biol. 1984;99(2):672–9. Epub 1984/08/01. doi: 10.1083/jcb.99.2.672. 6204996 PMC2113249

[24] Barton GJ. NOD: NucleOlar localization sequence Detector. Available from: http://www.compbio.dundee.ac.uk/www-nod/index.jsp. 10.1186/1471-2105-12-317 PMC3166288 21812952

[25] Tallini YN, Shui B, Greene KS, Deng KY, Doran R, Fisher PJ, Zipfel W, Kotlikoff MI. BAC transgenic mice express enhanced green fluorescent protein in central and peripheral cholinergic neurons. Physiol Genomics. 2006;27(3):391–7. Epub 2006/08/31. doi: 10.1152/physiolgenomics.00092.2006. 16940431

[26] Garcia-Cabezas MA, John YJ, Barbas H, Zikopoulos B. Distinction of Neurons, Glia and Endothelial Cells in the Cerebral Cortex: An Algorithm Based on Cytological Features. Frontiers in neuroanatomy. 2016;10:107. Epub 2016/11/17. doi: 10.3389/fnana.2016.00107. 27847469 PMC5088408

[27] Ludwig PED, Joe M. Histology, Glial Cells. Treasure Island, FL: StatPearls Publishing; 2023. 28722974

[28] Mitrea DM, Cika JA, Stanley CB, Nourse A, Onuchic PL, Banerjee PR, Phillips AH, Park CG, Deniz AA, Kriwacki RW. Self-interaction of NPM1 modulates multiple mechanisms of liquid-liquid phase separation. Nat Commun. 2018;9(1):842. Epub 2018/02/28. doi: 10.1038/s41467-018-03255-3. 29483575 PMC5827731

[29] Tesei G, Schulze TK, Crehuet R, Lindorff-Larsen K. Accurate model of liquid-liquid phase behavior of intrinsically disordered proteins from optimization of single-chain properties. Proc Natl Acad Sci U S A. 2021;118(44). Epub 2021/10/31. doi: 10.1073/pnas.2111696118. PMC8612223 34716273

[30] Ma K, Forbes JG, Gutierrez-Cruz G, Wang K. Titin as a giant scaffold for integrating stress and Src homology domain 3-mediated signaling pathways: the clustering of novel overlap ligand motifs in the elastic PEVK segment. J Biol Chem. 2006;281(37):27539–56. Epub 2006/06/13. doi: 10.1074/jbc.M604525200. 16766517

[31] Xu B, Zheng C, Chen X, Zhang Z, Liu J, Spencer P, Yang X. Dysregulation of Myosin Complex and Striated Muscle Contraction Pathway in the Brains of ALS-SOD1 Model Mice. ACS Chem Neurosci. 2019;10(5):2408–17. Epub 2019/03/21. doi: 10.1021/acschemneuro.8b00704. 30889949

[32] Kirby AJ, Palmer T, Mead RJ, Ichiyama RM, Chakrabarty S. Caudal-Rostral Progression of Alpha Motoneuron Degeneration in the SOD1(G93A) Mouse Model of Amyotrophic Lateral Sclerosis. Antioxidants (Basel). 2022;11(5). Epub 2022/05/29. doi: 10.3390/antiox11050983. PMC9137889 35624847

[33] Chio A, Logroscino G, Traynor BJ, Collins J, Simeone JC, Goldstein LA, White LA. Global epidemiology of amyotrophic lateral sclerosis: a systematic review of the published literature. Neuroepidemiology. 2013;41(2):118–30. Epub 2013/07/19. doi: 10.1159/000351153. 23860588 PMC4049265

[34] Ghasemi M, Brown RH Jr. Genetics of Amyotrophic Lateral Sclerosis. Cold Spring Harb Perspect Med. 2018;8(5). Epub 2017/03/09. doi: 10.1101/cshperspect.a024125. PMC5932579 28270533

[35] Mejzini R, Flynn LL, Pitout IL, Fletcher S, Wilton SD, Akkari PA. ALS Genetics, Mechanisms, and Therapeutics: Where Are We Now? Front Neurosci. 2019;13:1310. Epub 2019/12/24. doi: 10.3389/fnins.2019.01310. 31866818 PMC6909825

[36] Boylan K. Familial Amyotrophic Lateral Sclerosis. Neurol Clin. 2015;33(4):807–30. Epub 2015/10/31. doi: 10.1016/j.ncl.2015.07.001. 26515623 PMC4670044

[37] Ling SC, Polymenidou M, Cleveland DW. Converging mechanisms in ALS and FTD: disrupted RNA and protein homeostasis. Neuron. 2013;79(3):416–38. Epub 2013/08/13. doi: 10.1016/j.neuron.2013.07.033. 23931993 PMC4411085

[38] Neumann M, Sampathu DM, Kwong LK, Truax AC, Micsenyi MC, Chou TT, Bruce J, Schuck T, Grossman M, Clark CM, McCluskey LF, Miller BL, Masliah E, Mackenzie IR, Feldman H, Feiden W, Kretzschmar HA, Trojanowski JQ, Lee VM. Ubiquitinated TDP-43 in frontotemporal lobar degeneration and amyotrophic lateral sclerosis. Science. 2006;314(5796):130–3. Epub 2006/10/07. doi: 10.1126/science.1134108. 17023659

[39] Hardiman O, Al-Chalabi A, Chio A, Corr EM, Logroscino G, Robberecht W, Shaw PJ, Simmons Z, van den Berg LH. Amyotrophic lateral sclerosis. Nat Rev Dis Primers. 2017;3:17085. Epub 2017/10/21. doi: 10.1038/nrdp.2017.85. 29052611

[40] Mackenzie IR, Bigio EH, Ince PG, Geser F, Neumann M, Cairns NJ, Kwong LK, Forman MS, Ravits J, Stewart H, Eisen A, McClusky L, Kretzschmar HA, Monoranu CM, Highley JR, Kirby J, Siddique T, Shaw PJ, Lee VM, Trojanowski JQ. Pathological TDP-43 distinguishes sporadic amyotrophic lateral sclerosis from amyotrophic lateral sclerosis with SOD1 mutations. Ann Neurol. 2007;61(5):427–34. Epub 2007/05/01. doi: 10.1002/ana.21147. 17469116

[41] Vogler TO, Wheeler JR, Nguyen ED, Hughes MP, Britson KA, Lester E, Rao B, Betta ND, Whitney ON, Ewachiw TE, Gomes E, Shorter J, Lloyd TE, Eisenberg DS, Taylor JP, Johnson AM, Olwin BB, Parker R. TDP-43 and RNA form amyloid-like myo-granules in regenerating muscle. Nature. 2018;563(7732):508–13. Epub 2018/11/23. doi: 10.1038/s41586-018-0665-2. 30464263 PMC6324568

[42] Weihl CC, Temiz P, Miller SE, Watts G, Smith C, Forman M, Hanson PI, Kimonis V, Pestronk A. TDP-43 accumulation in inclusion body myopathy muscle suggests a common pathogenic mechanism with frontotemporal dementia. Journal of neurology, neurosurgery, and psychiatry. 2008;79(10):1186–9. Epub 2008/09/18. doi: 10.1136/jnnp.2007.131334. 18796596 PMC2586594

[43] Kotter S, Unger A, Hamdani N, Lang P, Vorgerd M, Nagel-Steger L, Linke WA. Human myocytes are protected from titin aggregation-induced stiffening by small heat shock proteins. J Cell Biol. 2014;204(2):187–202. Epub 2014/01/15. doi: 10.1083/jcb.201306077. 24421331 PMC3897184

[44] Bobylev AG, Galzitskaya OV, Fadeev RS, Bobyleva LG, Yurshenas DA, Molochkov NV, Dovidchenko NV, Selivanova OM, Penkov NV, Podlubnaya ZA, Vikhlyantsev IM. Smooth muscle titin forms in vitro amyloid aggregates. Biosci Rep. 2016;36(3). Epub 2016/04/30. doi: 10.1042/BSR20160066. PMC5293577 27129292

[45] Cheroni C, Marino M, Tortarolo M, Veglianese P, De Biasi S, Fontana E, Zuccarello LV, Maynard CJ, Dantuma NP, Bendotti C. Functional alterations of the ubiquitin-proteasome system in motor neurons of a mouse model of familial amyotrophic lateral sclerosis. Human molecular genetics. 2009;18(1):82–96. Epub 2008/10/02. doi: 10.1093/hmg/ddn319. 18826962 PMC3298865

[46] Rabin BA, Griffin JW, Crain BJ, Scavina M, Chance PF, Cornblath DR. Autosomal dominant juvenile amyotrophic lateral sclerosis. Brain. 1999;122 ( Pt 8):1539–50. Epub 1999/08/04. doi: 10.1093/brain/122.8.1539. 10430837

[47] Fang T, Jozsa F, Al-Chalabi A. Nonmotor Symptoms in Amyotrophic Lateral Sclerosis: A Systematic Review. Int Rev Neurobiol. 2017;134:1409–41. Epub 2017/08/15. doi: 10.1016/bs.irn.2017.04.009. 28805578

[48] Rubio MA, Herrando-Grabulosa M, Gaja-Capdevila N, Vilches JJ, Navarro X. Characterization of somatosensory neuron involvement in the SOD1(G93A) mouse model. Sci Rep. 2022;12(1):7600. Epub 2022/05/10. doi: 10.1038/s41598-022-11767-8. 35534694 PMC9085861

[49] Paxinos G, Franklin K.B.J. The Mouse Brain in Stereotaxic Coordinates. 2nd ed. San Diego: Academic Press; 2004.

[50] Livak KJ, Schmittgen TD. Analysis of relative gene expression data using real-time quantitative PCR and the 2(-Delta Delta C(T)) Method. Methods. 2001;25(4):402–8. Epub 2002/02/16. doi: 10.1006/meth.2001.1262. 11846609

[51] Warren CM, Krzesinski PR, Greaser ML. Vertical agarose gel electrophoresis and electroblotting of high-molecular-weight proteins. Electrophoresis. 2003;24(11):1695–702. Epub 2003/06/05. doi: 10.1002/elps.200305392. 12783444

